# Concerning Amputations1Read at the meeting of the Association of Wabash Railway Surgeons November 8, 1906. St. Louis, Mo.

**Published:** 1907-07

**Authors:** Miles F. Porter

**Affiliations:** Fort Wayne, Ind.; Surgeon to Hope Hospital; Professor of Surgery in the Indiana Medical College, Department of Medicine of Purdue University


### Concerning Amputations.[1]

By MILES F. PORTER, A.M., M.D., Fort Wayne, Ind.

Surgeon to Hope Hospital; Professor of Surgery in the Indiana Medical College, Department of Medicine of Purdue University.

[*Fort Wayne Medical Journal-Magazine, April, 1907.*]

I have taken the liberty of changing the title of my paper somewhat from that given in the program, as I have desired to give myself a little wider scope than would be indicated by that title.

When, where, and how to amputate, are the questions I desire to discuss. With an increase of knowledge, amputations

1 Read at the meeting of the Association of Wabash Railway Surgeons November 8, 1906. St. Louis, Mo.

have become more and more infrequent. Especially is this true of primary amputations. Indeed, as applied to parts of the hands or feet, primary amputations among really good surgeons may be placed among the rare operations. With proper antiseptic treatment the risk of waiting in doubtful cases is practically nil, hence it may be laid down as a rule that where there is the least chance of saving a more useful member than can be supplied by the maker of artificial limbs, amputation should not be done. In this connection it should be borne in mind that while it is possible to get a relatively useful artificial foot, the best artificial substitute for a hand is very poor. In rare cases, perhaps, the bad environment would cause one to decide in favor of amputation, but good hospital facilities are now practically always within reach, consequently the lack of them will seldom, if ever amount to an adequate excuse for doing a primary amputation in an otherwise questionable case.

The question when to amputate involves also the question as to the time of amputation, when it has been decided that amputation is necessary. When time is necessary to determine the viability of important tissues we should wait, but to wait for the subsidence of shock is worse than folly, for it adds to the discomfort of the patient, and to the risk to his life. To wait until a proper environment can be secured, by transportation or otherwise, for the proper conduct of the operation, is of course the part of wisdom.

In deciding the point of amputation (where to amputate) we should keep in mind the fact that an amputation is a sacrifice, and hence it is to be made as light for the patient as is possible. Again, in deciding this question we should remember that it is the character and quality of the tissue to be saved or sacrificed, rather than the quantity, that should concern us. To save an extremity or a part of an extremity that shall be to its possessor, during the remainder of his life, a thing of ugliness, and a curse, is to increase rather than mitigate the sacrifice.

With the improved technique of today a slight additional loss of tissue is so small a matter that it may be waived in favor of a stump better adapted to service, or to the wearing of an artificial limb.

An ideal amputation comprehends clean, straight cuts with a sharp knife, through undamaged tissues at an ideal point, retrenchment of nerves, perfect hemostasis and coaption, proper disposition of scar, and correct flap formation, all accomplished with perfect asepsis, a minimum amount of trauma, and in the least possible time consistent with perfect work. Such an amputation wound will and should be allowed to heal before changing the original dressing. To make flaps of damaged tissue with the consequent risk of sloughing or undue formation of cicatricial tissue is unwise except in some cases where length of stump is quite important, as, for instance, in injuries of the feet or hands where ofttimes locomotion or prehension is much improved by a slight addition in length of the stump. Irritable and painful scars are often due to unnecessary cicatricial formation, consequent upon the use of damaged tissue in the flaps. However, I do not want anything I have said on this subject to be taken as a warrant for unnecessary sacrifice of tissue, especially in hand injuries and diseases, and more especially where there is a loss of part of the member, making each bit of that which is left, exceptionally valuable to the patient.

In making finger flaps, it may be wise to shorten the member somewhat for the purpose of getting a long palmar flap, with an idea of preserving the tactile sensibility.

Drainage is an acknowledgement of imperfect work or materials, and hence, except in rare cases, inadvisable.

Sutures are sometimes necessary but should be dispensed with when not necessary, which is often the case. In a way we may say all sutures are bad, through and through sutures the worst, buried, absorbable sutures the best. Buried non-absorbable sutures left permanently are inexcusable. Ordinarily a very few sutures to coapt the muscles are all that are required. A very common error is the too tight tying of sutures. They should coapt, not strangulate. Very frequently the most perfect coaptation may be made by the proper adjustment of the dressings and adhesive without a single suture. The advantages of coaptation by adhesive and disposition of dressings, is that the coaptation thus secured is more perfect and yet not so tight as to interfere with the egress of secretions from the wound, while the trauma and infection-atria are reduced to a minimum, thus lessening the danger of infection. Moreover, this method of coaptation conduces to the comfort of the patient, and minimises the scar formation. This latter is of less importance in amputations than in surgery of the face and neck, but is worthy of note.

---

A New blood Test.—Max Einhorn, (*Medical Record,* June 8, 1907,) describes a new test for blood in the stomach contents, feces, and urine. It consists of testing with paper sensitised by the use of benzidin. The paper is immersed in the solution to be examined, and a few drops of peroxide of hydrogen are added, when a blue color is formed if blood is present. The blue or green color appears in a few seconds, and we should not wait more than a minute for the reaction to appear, as after that the color may appear from the presence of other substances than blood. HCl may cause the reaction after two or three minutes. The benzidin test can be recommended as a preliminary test: a strong reaction or no reaction at all gives a reliable test.

